# Application of Computational Methods for the Design of BACE-1 Inhibitors: Validation of *in Silico* Modelling

**DOI:** 10.3390/ijms15035128

**Published:** 2014-03-24

**Authors:** Marek Bajda, Jakub Jończyk, Barbara Malawska, Sławomir Filipek

**Affiliations:** 1 Faculty of Chemistry, University of Warsaw, Pasteura 1, 02-093 Warsaw, Poland; E-Mail: sfilipek@chem.uw.edu.pl; 2 Department of Physicochemical Drug Analysis, Faculty of Pharmacy, Medical College, Jagiellonian University, Medyczna 9, 30-688 Cracow, Poland; E-Mails: jakub.jonczyk@uj.edu.pl (J.J.); barbara.malawska@uj.edu.pl (B.M.)

**Keywords:** β-secretase, BACE-1, inhibitor, molecular modelling, docking studies, validation

## Abstract

β-Secretase (BACE-1) constitutes an important target for search of anti-Alzheimer’s drugs. The first inhibitors of this enzyme were peptidic compounds with high molecular weight and low bioavailability. Therefore, the search for new efficient non-peptidic inhibitors has been undertaken by many scientific groups. We started our work from the development of *in silico* methodology for the design of novel BACE-1 ligands. It was validated on the basis of crystal structures of complexes with inhibitors, redocking, cross-docking and training/test sets of reference ligands. The presented procedure of assessment of the novel compounds as β-secretase inhibitors could be widely used in the design process.

## 1. Introduction

β-Secretase (BACE-1), also called memapsin-2, is an important target for the development of anti-Alzheimer’s agents [1–3]. It belongs to the group of aspartyl proteases and is engaged in amyloidogenic pathway due to the cleavage of amyloid precursor protein (APP) at the β-site (Met-1–Asp1) [4]. BACE-1 splits APP into *N*-terminal, soluble domain sAPPβ and *C*-terminal fragment C99. A large sAPPβ fragment is released from the cells while C99 remains in the membrane and is then processed by γ-secretase to produce β-amyloid peptides (mainly, Aβ40 and Aβ42) [5]. Aβ aggregates, forming toxic oligomers [6]. Alzheimer’s disease is connected with overproduction of Aβ, which triggers a cascade of events leading to disruption in functioning of neurons [7]. Extracellular plaques accumulate nearby neurites and cause their degeneration [8].

The first reports of *BACE-1* gene knockout in mice revealed that the animals developed normally [9]. Further studies indicated that *BACE*^−/−^ mice displayed reduced synaptic plasticity and myelination, as well as deficiency in cognition and emotion performance test [10,11]. Nevertheless, β-secretase inhibitors have been proved to diminish a level of β-amyloid in the brain of mice and thus may be considered as a way of treating the disease, and not just alleviation of the symptoms [12]. Therefore, the search for novel BACE-1 inhibitors as potential therapeutics for Alzheimer’s disease (AD) has been undertaken by many scientists from both academia and the pharmaceutical companies [13–18].

β-Secretase acts on a wide diversity of substrates but in comparison with other aspartyl proteases, it prefers acidic or polar residues [19]. Apart from APP and its homologues, the enzyme also cleaves neuregulin and the β2 subunit of voltage-gated sodium channel, which both play an essential role in normal functioning of the brain [20,21]. BACE-1 is highly expressed in the brain and pancreas. Its recycling between endosomes and the trans-Golgi network is precisely controlled [22]. β-Secretase is synthesized as a 501 amino acid proenzyme and requires post-translational modifications. Pro-BACE-1 contains a signal peptide of 23 residues, followed by a 25 amino acid prosequence, a large catalytic domain with two conserved motifs, which are characteristic for aspartyl proteases, and a single transmembrane domain followed by a 23 amino acid cytosolic domain [19]. β-Secretase shares about 25% sequence homology with cathepsin D and renin, and has 64% similarity with its homologue BACE-2 (memapsin-1) [20]. BACE-2, in comparison with BACE-1, is located primarily in the heart, kidney, and placenta, with very low level in the brain [23].

**Figure 1.**
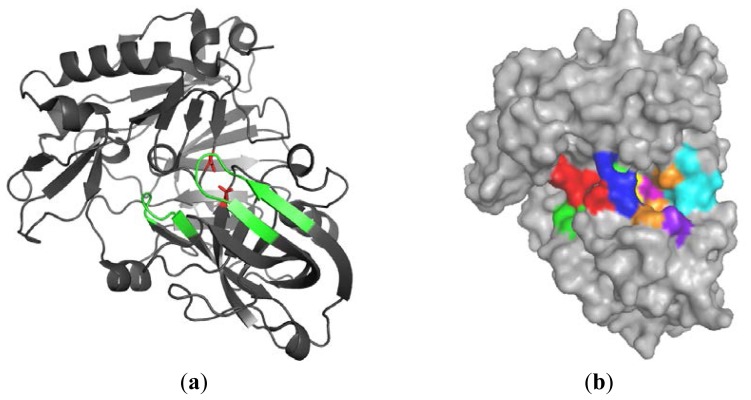
(**a**) Structure of β-secretase. The most important elements such as two catalytic aspartates, flap and 10s loop are highlighted in red and green; and (**b**) surface of the enzyme with selected subsites S4–S4′ (color codes: S4S3S2S1S1′S2′S3′S4′).

Many crystal structures of BACE-1, including the apo form, complexes with large-size peptic ligands and with rather small inhibitors, have been obtained (Figure 1) [24]. The first X-ray structure of the extracellular domain complexed with a peptide inhibitor OM99-2 was reported by Hong [25]. This compound possesses an unnatural hydroxyethylene linker which can mimic a transition state. The initial BACE-1 inhibitors such as OM99-2 were peptidomimetic compounds characterized by high molecular weight and their structure contained a secondary alcohol group that formed hydrogen bonds with the catalytic dyad. The enzyme catalytic domain is composed of two aspartyl protease motifs: DTGS and DSGT [26,27]. The active-site aspartate residues (Asp32 and Asp228) are co-planar and in apo form create H-bond network with a single water molecule between them. This water is responsible for initiation of nucleophilic attack on the peptide carbonyl group upon substrate binding [28]. The residues 67–77 occur in a β-hairpin conformation and are located over the active site, forming the flap [29]. The flap opens during the substrate entry, closes at the catalysis step and reopens to release the products of hydrolysis. Crystallographic studies revealed that peptidic inhibitors bind with β-secretase in a closed-flap form. Comparing the ligand free and ligand bound enzyme, it was observed that the flap adopts an open and a closed conformation, respectively. Residues 9–14 create a short loop between two β-strands at the base of the S3 pocket [30]. The conformation of this 10s loop also shows significant differences. BACE-1 also contains three disulfide bridges in the catalytic domain, which are essential for the correct folding and enzymatic activity [26,31].

According to Schlechter and Berger’s nomenclature, four residues on both sides of the cleavage site of APP are called P4–P4′ (P4, P3, P2, P1 ↓ P1′, P2′, P3′, P4′) [32], and the areas of enzyme which interact with substrate residues are named S4–S4′, respectively. Recently, the crystal structure of a potent inhibitor OM03-4, bound to β-secretase, revealed the presence of three extra subsites (S5–S7) [33,34]. The superimposition of BACE-1 and cathepsin D displays significant differences in the structure of the S1′/S3′ pocket and may be utilized to obtain the selectivity [24].

Significant efforts have been undertaken to identify low molecular weight, blood–brain barrier (BBB) penetrable, specific and stable non-peptide β-secretase inhibitors which could be developed as potential and effective anti-Alzheimer’s agents [35–37]. The aim of the presented studies was to propose a new *in silico* method for design and assessment of non-peptidic inhibitors of BACE-1. This method was based on docking procedure and validated on the basis of reference compounds from the literature data. Herein, we present the description and validation of the method.

## 2. Results and Discussion

The development of an effective method for the design of novel ligands requires assessment of this approach before it is widely used. In our case, we started from selection of the most suitable structure of BACE-1 for docking, which enables the best prediction of binding mode, and later we looked for the best scoring function to precisely predict the activity.

### 2.1. Analysis of Selected Crystal Structures

#### 2.1.1. β-Secretase (BACE-1)

**Figure 2.**
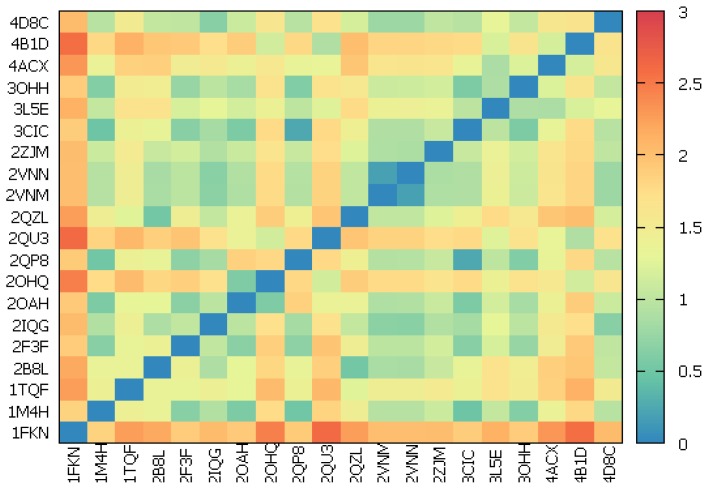
Matrix plot for root-mean-square deviation (RMSD) analysis. RMSD values are calculated for all heavy atoms of catalytic dyad, flap, 10s loop and residues within 8 Å from aspartates.

The Protein Data Bank (PDB) [38] currently contains almost 300 crystal structures of BACE-1. Among them, 20 high-resolution complexes (<2.11 Å) with potent and moderately potent, peptidic and non-peptidic inhibitors were selected for the analysis. As the ligand binding is dependent on the conformation of active site residues, special attention was paid to catalytic dyad (Asp32, Asp228), 10s loop composed of residues 9–14, flap consisting of amino acids 67–77 and all other residues within 8 Å from aspartates. The root-mean-square deviation (RMSD) values for all heavy atoms of such defined binding site ranged from 0.18 to 2.56 Å (Figure 2 and Table S1). Visual inspection showed the relative rigidity of almost whole selected residues except the amino acids building the flap and 10s loop, which had the largest contribution in RMSD values. The position of catalytic aspartates did not change in a significant way. The flap occurred in three different positions upon ligand binding. The closed conformation was dominant but close to open form (2OHQ, 2QU3, 4ACX, 4B1D) and transition form between these two (3L5E, 3OHH) also appeared. The 10s loop moved forward and backward to change the volume of active site and adopted one of a few positions with the most frequent position at the bottom. Comparison of crystal structures revealed no significant correlation between movements of the flap and 10s loop.

#### 2.1.2. Water Molecules in Crystal Structures

The water molecules in the vicinity of the catalytic dyad play an important role in the hydrolysis of peptide bonds by the β-secretase. It is also known that the presence of water affects the amount of hydrogen bonds which may occur between the ligand and amino acids in the enzyme active site. The analysis of 20 complexes included all waters present in the space within 8 Å from each ligand. It was noted that BACE-1 active center had contained from 15 to 57 solvent molecules, at the same time 0–8 waters interacted with the inhibitor (Table S2). There were eight crystal structures which comprised water interacting with at least one catalytic aspartate. The solvent molecules, which were found to create hydrogen bonds with the ligand, were later taken into account during validation of the docking procedure.

### 2.2. Validation of Docking with Gold Suite

#### 2.2.1. Redocking

In the first step of validation redocking, 20 previously mentioned complexes from PDB were used to check if Gold program [39] was able to reproduce original ligand poses. Hermes, the graphical user interface for Gold, was utilized to prepare the protein and to optimize the settings of docking. Seven hundred and twenty dockings, ten runs each, were performed. Three different sizes of binding site were tested due to the significant differences in the molecular volume of reference inhibitors. The active center was sequentially defined as all residues within 8, 10 and 12 Å from ligand molecule. In order to test the effect of water molecules, each docking was carried out with on, toggle and off options with regard to waters (Table S2). Four available scoring functions were evaluated to select the best poses. The results were analyzed and assessed in respect of run convergence and RMSD, which should receive the lowest value for the pose with the highest score.

The summary of redockings is presented in Table 1. The values of selected parameters were established for each complex to obtain the ligand pose which was the closest to the one in crystal. The shown settings of docking enabled us to get 10 complexes with good poses (RMSD ≤ 1.0 Å), 7 complexes with close poses (1 Å < RMSD ≤ 2 Å) and 3 with bad poses (RMSD > 3 Å). The worst poses were predicted for peptidic inhibitors from 1FKN, 1M4H and 1TQF crystal structures. It should be emphasized that Gold could not provide good or close poses for peptidic ligands of large flexibility, using basic functions. Application of constraints and interaction motifs was a solution for this problem but we did not want to introduce that kind of limitation. It was noted that GoldScore was the best scoring function in case of 13 complexes, and water was not necessary for docking in the case of 17 structures. Only in case of three complexes (2VNN, 3L5E, 4ACX), were RMSD values slightly better with water than without it. The different sizes of the active site were distributed similarly.

#### 2.2.2. Cross-Docking

**Figure 3.**
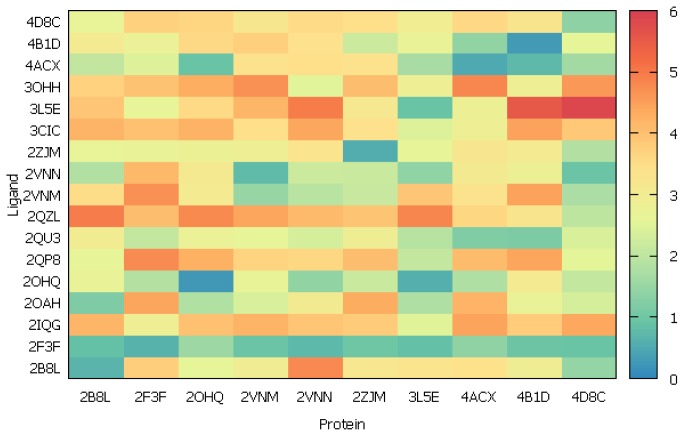
Matrix plot for RMSD analysis of ligand poses upon cross-docking.

According to the results of previous step, the enzyme structures from the top 10 complexes with the lowest RMSD value and all 17 non-peptidic inhibitors were selected for the cross-dockings. Each inhibitor was docked to the enzyme from each complex, using settings from Table 1 with respect to the protein. The quality of fit was evaluated on the basis of ligand RMSD values with the following ranges: RMSD ≤ 1.0 Å for good pose, 1 Å < RMSD ≤ 2 Å for close pose, 2 Å < RMSD ≤ 3 Å for pose with errors and RMSD > 3 Å for bad pose. The plot presenting the results of cross-docking is shown in the Figure 3 (see also Table S3).

It was noted that among 10 β-secretase structures, 4D8C and 3L5E were the most suitable for the docking due to the highest number of well adopted ligands. The first one, 4D8C, was BACE-1 with closed conformation of flap. It could adopt eight inhibitors, resulting in good and close poses. Moreover, the docking did not require any water molecules inside active center. Unfortunately, the ligands, which were naturally bound with unclosed flap BACE-1, docked to this structure with higher RMSD values, e.g., ligands from 3L5E or 3OHH. The second structure (3L5E) was the enzyme with transition state of the flap, and during the docking to this structure the water molecules were necessary to obtain the right binding mode. In this case, seven good and close poses were received.

Based on those findings, we selected the structure of β-secretase from 4D8C complex for further studies due to the close conformation of flap and docking without water.

#### 2.2.3. Scoring

It is very important to predict the activity correctly during the design of novel ligands. Therefore, we decided to examine the relationship between scoring function and the biological activity for a group of reference inhibitors. The ligand poses, in case of docking to the selected 4D8C structure, were finally assessed by GoldScore and we wanted to know if this scoring function was accurate enough or the results required rescoring. The previous set of non-peptidic ligands could not be used directly because it contained only 17 derivatives. Moreover, their activity was determined in different assays and should not be compared without standardization. Therefore, a novel group of 60 inhibitors was selected on the basis of literature data [40–45]. All compounds were tested in the assay, based on fluorescence resonance energy transfer (FRET) technique and using Rh-EVNLDAEFK-Quencher as substrate of BACE-1. They were structurally diverse and belonged to different chemical groups: aminopyridines, coumarines, flavonoids, diterpenoids, aminopropanoles, biaryls and fused-rings. The potency of these compounds was in a wide range of IC_50_ values: 56 nM–2 mM. (Table S4).

**Figure 4.**
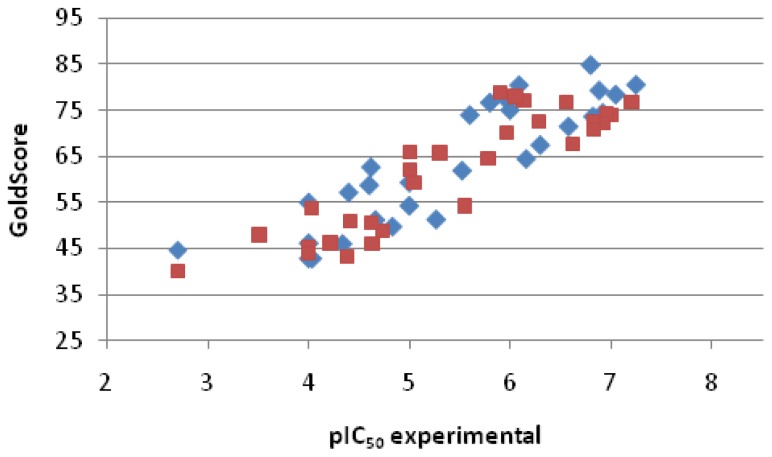
Plot of score *vs.* experimental pIC_50_ for the training and test set of reference inhibitors. Each set contains 30 compounds (blue marks: training set; and red marks: test set).

The prepared library of reference inhibitors was divided into two subgroups: training set and test set (Figure 4). Both sets of ligands were docked into β-secretase but the first one was used to establish a relationship between score and activity, expressed as pIC_50_; meanwhile, the second one was utilized to test the predictability of the potency by the method.

The statistically significant correlation between GoldScore and pIC_50_ was observed for the training set. Equation (1) describes this relationship. It is characterized by high correlation coefficient (*R* = 0.8822, *n* = 30).

(1) $${\text{pIC}}_{50}=0.0791\times \text{GoldScore}+0.410$$

**Figure 5.**
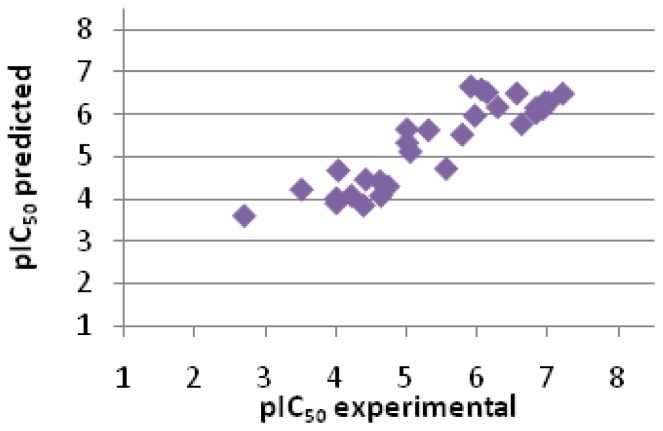
Correlation between predicted and experimental values of the activity for the test set (*R* = 0.8937, *n* = 30).

In the next step, the activity of the test set was predicted, based on GoldScore from docking and Equation 1, developed for the training set. It was observed that predicted values were highly correlated with experimental ones (*R* = 0.8937, *n* = 30, Figure 5 and Table 2), and the errors for prediction of pIC_50_ were really low for such kind of procedure. Indeed, the method enabled to differentiate compounds of high, moderate and low activity and can be used in the search of novel non-peptidic BACE-1 inhibitors. Docking to 4D8C β-secretase structure and assessment by GoldScore was especially advantageous because this scoring function gave accurate values and no rescoring was needed. The presented method could be useful in both virtual screening and fragment-based design of new potential BACE-1 inhibitors.

## 3. Experimental Section

### 3.1. Analysis of Selected Crystal Structures

The 20 crystal structures of human β-secretase were obtained from Protein Data Bank [38], and their codes are shown in Table 1. Mutual complex comparison was performed with PyMol 0.99rc2 (DeLano Scientific LLC., Palo Alto, CA, USA) [46] and Sybyl X 1.2 (Tripos, St. Louis, MO, USA) [47] programs. In case of oligomeric structure of BACE-1, one monomer was selected (Table 1) and used in further analyses. The interactions between enzyme, ligand and water in each complex were checked by LigX module, implemented in MOE 2009.10 (Chemical Computing Group, Montreal, QC, Canada) [48].

### 3.2. Docking and Scoring

The 3D structures of all ligands were created by Corina on-line [49] and saved as PDB files. Using Sybyl X 1.2, atom types were checked, protonation states were assigned, missing hydrogen atoms were added and Gasteiger-Marsili charges were assigned. Finally, the compound structures were saved in mol2 format.

During the protein preparation, all histidine residues were protonated at *N*ɛ, the hydrogen atoms were added and ligand molecules were removed. The binding site was sequentially defined as all amino acid residues within 8, 10 or 12 Å from original ligand. Some water molecules (specified in Table S2) were taken into account (on, toggle, off). Dockings were performed with Gold 5.1 (The Cambridge Crystallographic Data Centre, Cambridge, UK) [39]. A standard set of genetic algorithm with a population size of 100, a number of operations of 100,000 and clustering with a tolerance of 1 Å was applied. All scoring functions (GoldScore, ChemScore, ChemPLP, ASP) were tested for the evaluation of the docking results. For each ligand, the final results involved 10 poses, arranged on the ranking list, according to the scoring function values.

The best settings of docking to each selected crystal structure are listed in the Table 1.

## 4. Conclusions

Performed *in silico* analyses allowed us to propose the methodology for the design of novel non-peptidic potential inhibitors of β-secretase. It was fully validated and it was shown that the predictions were accurate. Based on redocking and cross-docking, among 20 crystal structures the most suitable complex was selected for the prediction of the binding mode. The structure 4D8C adopted many ligands correctly and the most importantly, the water molecules did not have to be taken into account during the docking. This made the method easier and faster. The group of 60 ligands, divided into two sets, enabled to check the relationship between the scoring function and the activity. The high correlation was found, and the regression equation was determined. Finally, it was proved that the method predicted the activity of test set precisely.

Further studies for verification this approach by synthesis of novel compounds and biological evaluation as potential BACE1 inhibitors are in progress.

## Supplementary Information



## Figures and Tables

**Table 1.** Optimal settings of Gold for redockings of 20 reference inhibitors.

Complex	Ligand *	Monomer (chain)	Binding site radius (Å)	Scoring function	Score value	RMSD	Water
1FKN	OM99-2	A	12	ChemPLP	101.86	3.94	no
1M4H	OM00-3	A	8	GoldScore	113.00	4.84	no
1TQF	32P	A	10	GoldScore	92.02	3.60	no
2B8L	5HA	A	12	GoldScore	133.76	0.65	no
2F3F	BDF488	C	12	ASP	54.23	0.31	no
2IQG	2FI	A	10	ASP	54.87	1.13	no
2OAH	QIN	A	8	ChemPLP	129.36	1.22	no
2OHQ	7IP	A	12	GoldScore	72.57	0.20	no
2QP8	SCH734723	A	8	GoldScore	97.51	1.43	no
2QU3	462	A	12	GoldScore	67.69	1.01	no
2QZL	IXS	A	10	GoldScore	116.81	1.15	no
2VNM	CM8	A	12	GoldScore	113.13	0.81	no
2VNN	CM7	A	8	ChemScore	52.00	0.47	yes
2ZJM	F1M	A	8	GoldScore	96.86	0.64	no
3CIC	SCH709583	A	10	GoldScore	114.20	1.27	no
3L5E	SCH736062	A	8	GoldScore	77.80	1.00	yes
3OHH	BMS681889	A	10	GoldScore	99.81	1.99	no
4ACX	S82	A	10	ChemPLP	99.61	0.51	yes
4B1D	6TG	A	10	ChemScore	32.00	0.27	no
4D8C	NVP-BXD552	C	10	GoldScore	102.69	0.95	no

* Original symbols of ligands were provided if available, otherwise symbols from PDB complexes.

**Table 2.** Comparison of predicted and experimental activities for the test set. Compounds are sorted by decreasing pIC_50_ experimental values.

Entry No.	GoldScore	pIC_50_ pred.	pIC_50_ exp.	Error
1	76.74	6.48	7.20	−0.72
2	74.07	6.27	7.00	−0.73
3	74.15	6.28	6.95	−0.68
4	72.20	6.12	6.92	−0.80
5	72.49	6.14	6.82	−0.68
6	70.94	6.02	6.82	−0.80
7	67.61	5.76	6.62	−0.86
8	76.82	6.49	6.55	−0.06
9	72.70	6.16	6.28	−0.12
10	77.07	6.51	6.13	0.38
11	78.03	6.58	6.05	0.53
12	70.10	5.96	5.96	0.00
13	78.88	6.65	5.90	0.75
14	64.45	5.51	5.78	−0.27
15	54.23	4.70	5.55	−0.85
16	65.78	5.61	5.30	0.31
17	59.32	5.10	5.05	0.06
18	66.03	5.63	5.00	0.63
19	61.99	5.31	5.00	0.31
20	48.90	4.28	4.73	−0.45
21	46.12	4.06	4.63	−0.57
22	50.47	4.40	4.62	−0.22
23	50.96	4.44	4.41	0.03
24	43.23	3.83	4.38	−0.55
25	46.15	4.06	4.21	−0.15
26	53.70	4.66	4.03	0.63
27	45.37	4.00	4.00	0.00
28	43.96	3.89	4.00	−0.11
29	47.96	4.20	3.51	0.70
30	40.10	3.58	2.70	0.88

pred. means predicted; and exp. means experimental.
