# Supplementary material for: Application of Computational Methods for the Design of BACE-1 Inhibitors: Validation of in Silico Modelling
Source: Int J Mol Sci. 2014 Mar 24;15(3):5128–39. doi: 10.3390/ijms15035128 (PMC3975444; doi:10.3390/ijms15035128)
Supplement: Supplementary file 1 [file ijms-15-05128-s001.pdf]

Supplementary Information

Table S1. Comparison of the β-secretase active site from different crystal structures: root-mean-square deviation (RMSD) for all heavy atoms (Å).

PDB	1FKN	1M4H	1TQF	2B8L	2F3F	2IQG	2OAH	2OHQ	2QP8	2QU3	2QZL	2VNM	2VNN	2ZJM	3CIC	3L5E	3OHH	4ACX	4B1D	4D8C
1FKN	0	1.82	2.25	2.17	1.88	2.03	1.91	2.45	1.90	2.60	2.25	2.00	2.00	2.01	1.88	2.11	1.89	2.29	2.56	2.03
1M4H	-	0	1.41	1.34	0.64	0.91	0.57	1.75	0.49	1.82	1.44	0.94	0.94	1.08	0.47	1.03	0.62	1.37	1.78	0.95
1TQF	-	-	0	1.34	1.32	1.41	1.31	2.02	1.39	2.06	1.25	1.44	1.44	1.51	1.39	1.67	1.49	1.85	2.11	1.50
2B8L	-	-	-	0	1.40	0.88	1.29	1.80	1.32	1.85	0.51	0.85	0.84	1.06	1.32	1.67	1.46	1.86	1.93	1.04
2F3F	-	-	-	-	0	1.02	0.65	1.86	0.67	1.95	1.45	0.97	0.98	1.14	0.64	1.17	0.73	1.46	1.91	1.01
2IQG	-	-	-	-	-	0	0.98	1.69	0.83	1.68	1.03	0.67	0.65	0.91	0.83	1.29	0.98	1.54	1.72	0.64
2OAH	-	-	-	-	-	-	0	0.58	1.85	1.37	1.37	0.88	0.91	1.07	0.57	1.14	0.83	1.39	1.88	1.09
2OHQ	-	-	-	-	-	-	-	0	1.78	1.14	1.88	1.75	1.75	1.64	1.76	1.39	1.67	1.56	1.13	1.60
2QP8	-	-	-	-	-	-	-	-	0	1.77	1.44	0.93	0.93	1.06	0.24	1.00	0.60	1.32	1.78	0.95
2QU3	-	-	-	-	-	-	-	-	-	0	1.95	1.82	1.83	1.73	1.76	1.24	1.66	1.32	0.90	1.68
2QZL	-	-	-	-	-	-	-	-	-	-	0	1.02	1.02	1.23	1.44	1.76	1.55	1.93	2.01	1.15
2VNM	-	-	-	-	-	-	-	-	-	-	-	0	0.18	0.89	0.90	1.39	1.10	1.62	1.80	0.77
2VNN	-	-	-	-	-	-	-	-	-	-	-	-	0	0.87	0.90	1.40	1.10	1.63	1.81	0.76
2ZJM	-	-	-	-	-	-	-	-	-	-	-	-	-	0	1.06	1.36	1.14	1.62	1.77	1.00
3CIC	-	-	-	-	-	-	-	-	-	-	-	-	-	-	0	0.98	0.57	1.32	1.76	0.94
3L5E	-	-	-	-	-	-	-	-	-	-	-	-	-	-	-	0	0.88	0.86	1.19	1.30
3OHH	-	-	-	-	-	-	-	-	-	-	-	-	-	-	-	-	0	1.21	1.63	1.03
4ACX	-	-	-	-	-	-	-	-	-	-	-	-	-	-	-	-	-	0	1.15	1.57
4B1D	-	-	-	-	-	-	-	-	-	-	-	-	-	-	-	-	-	-	0	1.65
4D8C	-	-	-	-	-	-	-	-	-	-	-	-	-	-	-	-	-	-	-	0

Table S2. Water molecules, present in the active site of β-secretase. The active center was defined as the space within 8 Å from the ligand. The numbers of waters correspond to the number from Protein Data Bank (PDB) files. Water molecules written in **bold** interact with ligand and were taken into account during validation of docking. The numbers highlighted in red indicate water molecules that interact with catalytic aspartates.

1FKN	1M4H	1TQF	2B8L	2F3F	2IQG	2OAH	2OHQ	2QP8	2QU3	2QZL	2VNM	2VNN	2ZJM	3CIC	3L5E	3OHH	4ACX	4B1D	4D8C
**9**	**15**	404	393	607	903	906	408	918	464	451	2008	2008	450	602	517	416	2012	2003	603
**10**	**52**	**405**	394	608	908	913	409	919	466	452	2009	2009	458	631	840	421	2013	2007	605
**11**	**85**	425	399	609	909	916	411	920	471	454	2011	2011	461	673	841	425	2015	2008	608
**12**	**189**	431	406	614	912	918	412	**921**	474	459	2012	2012	462	676	842	434	2027	2011	**617**
**13**	213	433	421	623	925	924	441	922	483	475	2031	2032	463	689	843	439	2032	2012	624
388	**224**	**436**	424	652	**928**	942	444	923	486	483	2033	2033	478	705	**844**	452	**2033**	**2013**	625
391	**225**	438	**429**	658	929	943	468	924	**487**	486	2050	**2034**	486	720	845	462	2035	2014	626
401	387	449	430	**666**	931	**944**	514	925	490	**489**	2051	2051	502	731	848	**476**	2036	2015	627
414	390	450	433	685	943	947	567	926	491	497	2052	2072	529	746	849	480	2037	2029	630
430	393	451	436	695	966	969	575	927	504	501	2083	2085	583	768	850	481	2038	**2030**	632
442	**398**	458	441	697	968	972	582	928	509	502	2086	2088	611	770	852	497	2039	2031	**647**
446	399	459	448	698	983	966	593	929	515	504	2087	2089	613	772	853	**502**	2044	2032	648
451	409	460	458	700	1000	988	595	930	533	506	2088	2090	614	775	854	533	**2080**	2033	649
462	**419**	482	470	710	1002	1017	605	931	554	526	2089	2091	618	834	**855**	538	**2082**	2036	653
473	424	**489**	**474**	716	1004	1018	621	932	571	528	2091	2094	624	856	856	577	2104	2042	660
482	439	492	476	718	1009	1026	-	933	574	534	2092	2095	625	881	858	**578**	2105	2064	666
487	440	495	483	726	1017	1033	-	934	-	535	2093	2096	635	939	859	592	2108	2066	676
**490**	446	496	504	734	1026	1038	-	935	-	544	2094	2097	639	978	860	**594**	2150	2068	679
502	456	509	**519**	742	1052	1058	-	936	-	551	2125	2134	644	990	861	**595**	2169	2072	686
507	457	510	521	768	1066	1080	-	937	-	555	2129	2136	645	1016	862	596	2170	2095	697

Table S2. *Cont.*

1FKN	1M4H	1TQF	2B8L	2F3F	2IQG	2OAH	2OHQ	2QP8	2QU3	2QZL	2VNM	2VNN	2ZJM	3CIC	3L5E	3OHH	4ACX	4B1D	4D8C
514	468	525	538	-	1072	1085	-	938	-	**565**	2133	2140	648	-	863	603	**2171**	2097	698
520	497	529	539	-	1085	1101	-	939	-	581	2204	2144	662	-	864	607	2172	2098	705
537	509	**537**	545	-	1094	1122	-	941	-	588	2229	2145	668	-	865	622	2173	2146	**719**
540	512	543	557	-	1097	1134	-	942	-	595	2230	2158	674	-	867	**633**	2174	2147	720
560	530	548	561	-	1108	1140	-	943	-	596	**2231**	**2162**	686	-	869	640	2178	**2171**	732
589	534	558	576	-	1110	1146	-	944	-	**604**	2232	2221	688	-	870	641	2179	2172	**733**
599	554	559	580	-	1121	**1155**	-	946	-	**608**	**2233**	2246	705	-	871	653	2247	2173	749
560	570	562	585	-	1122	1162	-	947	-	623	2326	2247	-	-	872	670	-	2177	750
636	575	566	-	-	1123	1164	-	948	-	**625**	**2327**	2248	-	-	873	674	-	2252	768
641	586	569	-	-	1124	1165	-	950	-	631	2329	2249	-	-	874	681	-	-	769
643	593	584	-	-	1163	1173	-	951	-	637	2333	2250	-	-	875	**683**	-	-	788
645	-	599	-	-	1165	1182	-	952	-	656	2334	2251	-	-	876	684	-	-	798

Table S3. Results of cross-dockings to ten crystal structures of β-secretase (BACE-1): Assessment with RMSD values. Asterisks indicate complexes which required water molecules for better results of docking.

Ligand	Protein									
	**2B8L**	**2F3F**	**2OHQ**	**2VNM ***	**2VNN**	**2ZJM**	**3L5E ***	**4ACX ***	**4B1D**	**4D8C**
2B8L	0.65	3.74	2.56	2.93	4.78	3.13	3.28	3.41	2.91	1.44
2F3F	0.85	0.63	1.53	0.91	0.72	0.98	0.83	1.38	0.93	0.90
2IQG	4.16	2.85	3.95	4.19	3.89	3.78	2.50	4.43	3.77	4.36
2OAH	1.16	4.39	1.81	2.36	3.01	4.30	1.79	4.19	2.68	2.31
2OHQ	2.69	1.84	0.24	2.66	1.41	2.13	0.57	1.79	3.03	2.06
2QP8	2.61	4.74	4.24	3.64	3.58	4.03	2.07	4.06	4.40	2.55
2QU3	2.96	2.06	2.73	2.63	2.32	2.91	1.88	1.17	1.15	2.38
2QZL	4.93	4.02	4.76	4.40	4.09	3.92	4.82	3.59	3.25	1.98
2VNM	3.48	4.66	3.03	1.48	1.92	2.12	3.87	3.34	4.44	1.72
2VNN	1.82	4.10	3.08	0.76	2.18	2.13	1.38	3.05	2.82	0.92
2ZJM	2.67	2.69	2.78	2.85	3.28	0.55	2.59	3.18	3.04	1.83
3CIC	4.18	3.96	4.20	3.43	4.38	3.38	2.42	2.86	4.44	3.83
3L5E	3.88	2.59	3.52	4.15	4.94	3.10	0.89	2.83	5.53	5.84
3OHH	3.67	3.92	4.28	4.66	2.52	4.02	2.85	4.80	2.86	4.58
4ACX	2.05	2.42	0.90	3.35	3.43	3.35	1.69	0.48	0.72	1.62
4B1D	3.03	2.72	3.56	3.72	3.38	2.19	2.69	1.40	0.27	2.58
4D8C	2.66	3.68	3.60	3.16	3.51	3.42	2.92	3.56	3.25	1.34

Table S4. The library of compounds, containing sixty inhibitors, which was used to examine scoring–activity relationship (S, training set; T, test set; and *, numbers of compounds in the original papers).

Entry No.	Set	Publication	No. (ref.) *	IC_{50} exp. [μM]	pIC_{50} exp.	GoldScore
1	S	Congreve *et al.* *J. Med. Chem.* **2007**, *50*, 1124–1132 [40]	**2**	2000	2.70	44.7
2	S		**8a**	0.69	6.16	64.57
3	T		**8b**	5	5.30	65.78
4	T		**7**	9	5.05	59.32
5	S		**6c**	24	4.62	62.79
6	S		**4**	25	4.60	58.87
7	S		**6b**	40	4.40	57.29
8	T		**3**	94	4.03	53.7
9	S		**6a**	100	4.00	55.13
10	T		**5**	310	3.51	47.96
11	T		**1**	2000	2.70	40.1
12	T	Piazzi *et al.* *Bioorg. Med. Chem Lett.* **2008**, *18*, 423–426 [41]	**3**	0.1	7.00	74.07
13	S		**8**	0.11	6.96	73.92
14	S		**4**	0.12	6.92	74.54
15	S		**5**	0.13	6.89	79.4
16	T		**2**	0.15	6.82	72.49
17	T		**1**	0.24	6.62	67.61
18	T		**7**	0.12	6.92	72.2
19	S		**6**	0.15	6.82	73.76
20	T		**9**	0.151	6.82	70.94
21	T	Shimmyo *et al.* *Biochim. Biophys. Acta* **2008**, *1780*, 819–825 [42]	**Myr**	2.8	5.55	54.23
22	S		**Que**	5.4	5.27	51.45
23	S		**Kae**	14.7	4.83	49.91
24	S		**Mor**	21.7	4.66	51.34
25	T		**Api**	38.5	4.41	50.96

Table S4. *Cont.*

Entry No.	Set	Publication	No. (ref.) *	IC_{50} exp. [μM]	pIC_{50} exp.	GoldScore
26	T	Jung *et al.* *Arch. Pharm. Res.* **2009**, *32*, 1399–1408 [43]	**13**	100	4.00	45.37
27	S		**4**	100	4.00	43
28	T		**5**	100	4.00	43.96
29	S		**3**	100	4.00	46.3
30	T		**10**	18.58	4.73	48.9
31	T		**11**	23.4	4.63	46.12
32	T		**9**	24.1	4.62	50.47
33	T		**2**	41.32	4.38	43.23
34	S		**12**	46.09	4.34	46.15
35	T		**1**	61.21	4.21	46.15
36	S		**8**	92.2	4.04	42.99
37	S	Asso *et al.* *ChemMedChem* **2008**, *3*, 1530–1534 [44]	**2g**	10	5.00	59.46
38	T		**2f**	10	5.00	66.03
39	S		**2d**	10	5.00	54.42
40	T		**2c**	10	5.00	61.99
41	S		**2b**	0.5	6.30	67.59
42	T		**1**	1.1	5.96	70.1
43	T		**2e**	1.66	5.78	64.45
44	S		**2a**	2.99	5.52	62.03

Table S4. *Cont.*

Entry No.	Set	Publication	No. (ref.) *	IC_{50} exp. [μM]	pIC_{50} exp.	GoldScore
45	S		**5c**	0.056	7.25	80.65
46	T		**5b**	0.063	7.20	76.74
47	S		**6c**	0.089	7.05	78.53
48	T		**6b**	0.112	6.95	74.15
49	S		**4b**	0.158	6.80	84.94
50	S		**5e**	0.262	6.58	71.62
51	T	Chirapu *et al.* Bioorg. *Med. Chem. Lett.* **2009**, *19*, 264–274 [45]	**1**	0.281	6.55	76.82
52	T		**5a**	0.524	6.28	72.7
53	T		**5d**	0.740	6.13	77.07
54	S		**3b**	0.811	6.09	80.57
55	T		**2c**	0.889	6.05	78.03
56	S		**6a**	0.998	6.00	75.15
57	S		**3c**	1.119	5.95	77.71
58	T		**3d**	1.256	5.90	78.88
59	S		**2a**	1.581	5.80	76.79
60	S		**2b**	2.506	5.60	74.09

exp. means experimental.

© 2014 by the authors; licensee MDPI, Basel, Switzerland. This article is an open access article distributed under the terms and conditions of the Creative Commons Attribution license (http://creativecommons.org/licenses/by/3.0/).
